# Chikungunya virus outbreak in Sint Maarten, 2013–2014

**DOI:** 10.26633/RPSP.2017.61

**Published:** 2017-07-04

**Authors:** Maria Henry, Lorraine Francis, Virginia Asin, Karen Polson-Edwards, Babatunde Olowokure

**Affiliations:** 1 General Health Care Section Department of Collective Prevention Services, Ministry of Public Health, Social Development and Labour Philipsburg Sint Maarten General Health Care Section, Department of Collective Prevention Services, Ministry of Public Health, Social Development and Labour, Philipsburg, Sint Maarten.; 2 Surveillance, Disease Prevention and Control Caribbean Public Health Agency Port-of-Spain Trinidad and Tobago Surveillance, Disease Prevention and Control, Caribbean Public Health Agency, Port-of-Spain, Trinidad and Tobago.

**Keywords:** Chikungunya virus, chikungunya fever, communicable diseases, emerging, Caribbean Region; Americas, Virus Chikungunya, fiebre chikungunya, enfermedades transmisibles emergentes, Region del Caribe, Americas

## Abstract

This report describes the outbreak of chikungunya virus (CHIKV) in Sint Maarten, a constituent country of Kingdom of the Netherlands comprising the southern part of the Caribbean island of Saint Martin, from 22 December 2013 (first reported case) through 5 December 2014. The outbreak was first reported by the French overseas collectivity of Saint-Martin in the northern part of the island—the first site in the Americas to report autochthonous transmission of CHIKV. By 5 December 2014, Sint Maarten had reported a total of 658 cases—an overall attack rate of 1.76%. Actual prevalence may have been higher, as some cases may have been misdiagnosed as dengue. Fever and arthralgia affected 71% and 69% of reported cases respectively. Of the 390 laboratory-confirmed cases, 61% were female and the majority were 20–59 years old (mean: 42; range: 4–92). The spread of CHIKV to Sint Maarten was inevitable given the ease of movement of people, and the vector, island-wide. Continuing their history of collaboration, the French and Dutch parts of the island coordinated efforts for prevention and control of the disease. These included a formal agreement to exchange epidemiological information on a regular basis and provide alerts in a timely manner; collaboration among personnel through joint island-wide planning of mosquito control activities, especially along borders; notification of all island visitors, upon their arrival at airports and seaports, of preventative measures to avoid being bitten by mosquitoes; dissemination of educational materials to the public; and island-wide public awareness campaigns, particularly in densely populated areas, for both residents and visitors. The information provided in this report could help increase understanding of the epidemiological characteristics of CHIKV and guide other countries dealing with vector-borne epidemics.

## RESUMEN

En el presente artículo se describe el brote del virus del chikungunya (CHIKV) que tuvo lugar entre el 22 de diciembre del 2013 (primer caso notificado) y el 5 de diciembre del 2014 en Sint Maarten, uno de los países integrantes del Reino de los Países Bajos, que comprende la parte sur de la isla caribeña de San Martín. El brote fue notificado primero por la colectividad de ultramar francesa de Saint-Martin, que ocupa la parte norte de la isla, convirtiéndose en la primera zona de las Américas en describir la transmisión autóctona del CHIKV. El 5 de diciembre del 2014, Sint Maarten había notificado 658 casos, equivalentes a una tasa de ataque del 1,76%, si bien la prevalencia real quizá haya sido mayor, puesto que algunos casos pueden haberse confundido por dengue. El 71% y el 69% de los casos notificados cursaron con fiebre y artralgias, respectivamente. De los 390 casos confirmados por laboratorio, el 61% eran mujeres y la mayoría tenían entre 20 y 59 años de edad (media: 42; intervalo: 4-92). Era inevitable que el CHIKV pasara a Sint Maarten, dadas la facilidad de movimiento de las personas y la extensión del vector por toda la isla. Siguiendo la tradición de cooperación mutua, las partes francesa y holandesa de la isla coordinaron las actuaciones de prevención y control, que consistieron en: la formalización de un convenio para intercambiar datos epidemiológicos de forma regular y emitir alertas puntualmente; la colaboración del personal de uno y otro lado para planificar, en todo el territorio insular, las actividades de control de mosquitos, sobre todo a lo largo de las fronteras; la notificación a todos los viajeros en arribo, a su llegada a los puertos y aeropuertos, de las medidas preventivas para evitar la picadura de los mosquitos; la difusión de materiales didácticos; y la realización de campañas públicas de concientización por toda la isla, en particular en las áreas de mayor densidad demográfica, dirigidas tanto a residentes como a turistas.

La información expuesta en este informe puede ayudar a conocer mejor las características epidemiológicas del CHIKV y servir de orientación para otros países que hagan frente a epidemias transmitidas por vectores.

Chikungunya virus (CHIKV) is a mosquito-borne virus (Alphavirus genus, *Togaviridae* family). First isolated in Tanzania in 1953, the virus has been identified in well-documented outbreaks in Africa, Asia, and the Pacific (1). Cases of CHIKV have also been reported in northern Italy (2007), Thailand (2009), the Democratic Republic of Congo (2011), and Cambodia (2012) (2). CHIKV is transmitted by the bite of *Aedes* mosquitoes— mainly *A. aegypti* ****and *A. albopictus.* Typical clinical signs of the disease include fever and severe arthralgia, which may persist for weeks, months, or years (3). General complications include myocarditis, hepatitis, and ocular and neurological disorders (2). Detection and diagnosis of the disease can be challenging, especially in settings where dengue is endemic.

The first evidence of autochthonous transmission of CHIKV in the Americas was reported to have occurred in the French overseas collectivity of Saint-Martin, the northern 60% of the Caribbean island of Saint Martin. The report was submitted on 6 December 2013 by the French National Reference Laboratory, (Regional Health Agency *(Agence Regionale de Santé,* ARS) of Guadeloupe, Saint-Martin, and Saint Barthélemy (“St. Barts”), Hope Estate, Saint-Martin). The infection occurred in the district of Oyster Pond, which overlaps with Sint Maarten, the Dutch constituent country that comprises the southern part of the island (4). Sint Maarten reported its first case of CHIKV on 22 December 2013 (5).

Dengue is endemic to the island, and although a dengue epidemic was ongoing at the time, negative laboratory tests suggested a cause other than dengue virus.

This report describes the outbreak of CHIKV in Sint Maarten through 5 December 2014. The information provided below could help increase understanding of the epidemiological characteristics of this emerging infectious disease and guide other countries dealing with vector-borne epidemics.

## MATERIALS AND METHODS

Surveillance for CHIKV cases in Sint Maarten began on 8 December 2013, two days after the first suspected cases were reported in the French part of the island. Syndromic surveillance is well-established in Sint Maarten, with seven sentinel sites throughout the country reporting cases of undifferentiated fever (UDF) to the General Health Care Section of the Department of Collective Prevention Services at the Ministry of Public Health, Social Development and Labour *(Ministerie van Volksgezondheid, Sociale Ontwikkeling en Arbeid Zaken, *VSA) in Philipsburg. The cases were then investigated for chikungunya infection.

CHIKV case data were collected using an epidemiological survey form with questions about demographic information, clinical features, contact history, and travel history. The case definition used for detecting and reporting suspected cases was acute fever (> 38.5°C) accompanied by joint pain, with no other aetiology for the joint pain (3). Laboratory confirmation required demonstration of a positive polymerase chain reaction (PCR) or a positive virus-specific immunoglobulin M (IgM) enzyme-linked immunosorbent assay (ELISA) antibody test (3). Dengue is endemic to the island and both Saint-Martin and Sint Maarten have the capacity to perform dengue quantitative reverse transcriptase real-time PCR (RT-qPCR) and serology (IgM) testing to support differential diagnoses. Although a dengue epidemic was ongoing at the time, negative laboratory tests suggested a cause other than dengue virus. CHIKV was detected in the three cases that tested negative for dengue by the ARS of Guadeloupe, Saint-Martin, and St. Barts, using RT-qPCR. The date of onset of illness and travel history were not available. Subsequent samples from suspected cases were tested at the Netherlands National Institute for Public Health and the Environment (*Rijksinstituut voor Volksgezondheid en Milieu*, RIVM) (Bilthoven, Kingdom of the Netherlands). Data were summarized using descriptive statistics from analyses performed on a database created using Microsoft Excel (Microsoft, Redmond, Washington, United States).

## RESULTS

No significant rise in reported cases of UDF was noted in national surveillance data during December 2013. However, an increase in reported cases was detected for epidemiological week 5 (EW5) of 2014 (week beginning 26 January 2014). From 6 December 2013 to 5 December 2014, a total of 658 chikungunya cases were reported, for an overall attack rate of 1.76% (population 37 427; 2011 census). Of these, 390 were laboratory confirmed, with 272 (70%) confirmed by RT-qPCR and 118 (30%) by serologic methods. No concurrent cases of dengue and CHIKV were detected during that period.

**FIGURE 1.**
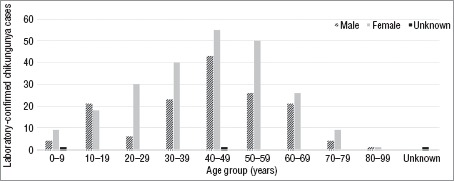
Distribution of age and sex for laboratory-confirmed cases of chikungunya virus, Sint Maarten, 6 December 2013–5 December 2014

***Source:*** Prepared by the authors based on the study results.

Of the 390 confirmed cases, 238 (61%) were female and 149 (38%) were male. Three cases (1%) did not have data on gender. Patients’ mean age was 42 years (range: 4–92) and the largest proportion of them (99 or 25%) were in the 40–49 year age group (Figure 1).

No fatalities associated with chikungunya infection were reported during this period. Major clinical characteristics experienced by patients were fever (71%) and arthralgia (69%). Rash was experi-enced by 19% of patients.

## DISCUSSION

Sint Maarten’s well-established syn-dromic surveillance system, which includes seven sentinel sites countrywide, reports data to the VSA’s Department of Collective Prevention Services. The surveillance data showed an increase in UDF beginning in EW5 2014 compared to the four-week 2010–2013 average. Historically, fewer than five cases of UDF per week were reported during 2010–2013, compared to 1–23 cases during 2014. Despite the concurrent outbreak of dengue, the increase in reported cases of UDF was likely due to CHIKV, as laboratory tests for dengue were negative.

The CHIKV attack rate in Sint Maarten (1.76%) was much lower than that of other documented outbreaks (6, 7). Some Sint Maarten residents with UDF symptoms (suspected CHIKV) were diagnosed by physicians in the French part of the island, which may have skewed this rate. Residents from Sint Maarten have access to health care services in Saint-Martin and vice versa. There is no physical or ecological barrier separating the French and Dutch parts of the island, enabling the free movement of people and vectors. The two countries have a history of collaborating to address common issues and this cooperation was evident with the 2014 signing of a Declaration of Intent to work together to combat CHIKV (8). Officials from both countries agreed to exchange epidemiological information on CHIKV on a regular basis and to communicate in a timely manner about any abnormal event related to the disease or its vector.

Other potential reasons for the lower CHIKV attack rate found in Sint Maarten include the fact that 3%’25% of cases could have been asymptomatic, and the likelihood that only those with more severe symptoms—or the “worried-well” (people who are nervous about their health)—would have sought out health services (9). Another potential cause of possible underestimation of the CHIKV attack rate was the concurrent dengue outbreak. Concurrent outbreaks of dengue and CHIKV pose clinical and diagnostic difficulties because the clinical symptoms are similar (10). For example, even though arthritis is regarded as the pathognomonic sign of chikungunya infection, those presenting with a mild case of it may have been misdiagnosed as dengue.

During the same period (December 2013–2014), a total of 793 confirmed cases of CHIKV were reported by Saint-Martin. As the two countries have similar population sizes, the lower number of confirmed cases reported by Sint Maarten is likely due to limited diagnostic capacity and underreporting (3).

As reported in other CHIKV outbreaks, there were more reported cases among females than males in most age groups. This may be due to greater health-seeking behavior, differing levels of exposed skin, and greater exposure due to peridomestic activities among women versus men (7).

The largest proportion of confirmed CHIKV cases (25%) were in the 40–49 year age group, and 61% of those oc-curred within patients 30–59 years old, suggesting both short- and long-term economic effects from the disease, including a drop in workplace productivity due to absenteeism, as a result of disease sequelae. Another possible effect, given that symptoms may persist for weeks, months, or years, is an increased burden on health and social services.

In response to the outbreak, risk communication messages were disseminated via print and audio media and made available in airports and seaports. Vector control for *A. aegypti* is routinely performed in Sint Maarten and consists of the removal of breeding sites, application of larvacides, and fogging. Vector control activities were intensified following recommendations from a joint Caribbean Public Health Agency (CARPHA) and Pan American Health Organization (PAHO) field support and outbreak response mission to Sint Maarten 7–11 January 2014. The authorities of both the French and Dutch parts of the island agreed to enhance their mosquito control programs and staff to ensure effective vector control. This collaboration among personnel of the two countries was evident in the joint planning of island-wide mosquito control activities, especially along borders. Collective communication materials were jointly prepared for dissemination to the wider public, and a Coordinating Committee comprising representatives from several disciplines from both countries was established in the event of a serious health threat or epidemic risk of mosquito-borne diseases.

A number of island-wide public awareness campaigns targeting both residents and visitors were carried out, with a focus on densely populated areas, and included posting flyers and banners and disseminating public service announcements. Upon their arrival to the island, passengers were urged to take preventive measures to avoid being bitten by mosquitoes, such as using mosquito repellent on exposed skin, and wearing long-sleeve shirts and pants/skirts, especially during dawn and dusk. Other preventative measures for preventing mosquito bites were posted on monitors at the Princess Juliana International Airport. In addition, at the Wathey Cruise Facility in Sint Maarten, mosquito repellent was provided at point-of-entry information desks, stores, and restaurants, and by taxi drivers and tour bus and water taxi operators. General practitioners (GPs) across Sint Maarten were provided with information regarding the disease, and surveillance activities by the VSA were enhanced. Neighboring overseas territories of the Netherlands— the islands of Bonaire, Sint Eustatius, and Saba (BES)—were informed of the emerging epidemic and advised to intensify surveillance for CHIKV.

The introduction and spread of CHIKV in Sint Maarten could not have been prevented due to 1) the high level of mobility of island residents and visitors throughout French Saint-Martin and Dutch Sint Maarten and 2) the ubiquitous nature of the *A. aegypti *vector. The two countries shared epidemiological information on the disease on a routine basis, strengthened their diagnostic capacity, and collaborated on joint vector control activities and public awareness/communication strategies.

This outbreak demonstrated the importance of technical cooperation between countries and the need for innovative prevention and control strategies for reducing the health risks linked to CHIKV and other arboviruses.

## Acknowledgments.

The authors thank the staff of the Surveillance and Vector Teams of the General Health Care Section of Collective Prevention Services at the Ministry of Public Health, Social Development and Labour; the Physician Base Sentinels; Sint Maarten Laboratory Services; Sint Maarten Medical Services; local practicing physicians; American University of the Caribbean; St. Maarten Justice Academy; the local population of St. Maarten; and the Caribbean Public Health Agency for their contributions to this work.

## Disclaimer.

Authors hold sole responsibility for the views expressed in the manuscript, which may not necessarily reflect the opinion or policy of the *RPSP/ PAJPH* or the Pan American Health Organization (PAHO).
